# The Key Role of Lifestyle Factors in Perpetuating Chronic Pain: Towards Precision Pain Medicine

**DOI:** 10.3390/jcm11102732

**Published:** 2022-05-12

**Authors:** Jo Nijs, Felipe Reis

**Affiliations:** 1 Pain in Motion Research Group (PAIN), Department of Physiotherapy, Human Physiology and Anatomy, Faculty of Physical Education and Physiotherapy, Vrije Universiteit Brussel, 103 Laarbeeklaan, 1090 Brussels, Belgium; felipe.reis@ifrj.edu.br; 2 Department of Physical Medicine and Physiotherapy, University Hospital Brussels, 1090 Brussels, Belgium; 3 Unit of Physiotherapy, Department of Health & Rehabilitation, Institute of Neuroscience and Physiology, University of Gothenburg, 405 30 Gothenburg, Sweden; 4 Physical Therapy Department, Instituto Federal do Rio de Janeiro (IFRJ), Rio de Janeiro 20270-021, Brazil; 5 Postgraduation Program-Clinical Medicine Department of Universidade Federal do Rio de Janeiro (UFRJ), Rio de Janeiro 21941-901, Brazil

## 1. Introduction

Chronic pain has a massive personal and socioeconomic impact and remains a challenge for many clinicians around the world. Cumulating evidence shows that lifestyle factors such as physical (in)activity, stress, poor sleep, unhealthy diet, and smoking are associated with chronic pain severity and perpetuate the condition across all age categories [1]. Precision medicine is defined as the ability to classify patients into subgroups that differ in their susceptibility to biology, or prognosis of a particular disease, or in their response to a specific treatment, and thus to tailor treatment to the individual patient characteristics [2]. A paradigm shift from a tissue- and disease-based approach towards individually tailored multimodal lifestyle interventions holds great potential for improved outcomes and decrease the psychological and socioeconomic burden of chronic pain. Such a multimodal lifestyle approach fits well into the global move towards precision pain medicine for patients with chronic pain [3]. For all these reasons, this Special Issue of *Journal of Clinical Medicine* is dedicated to Lifestyle and Chronic Pain, and the paradigm shift towards a multimodal lifestyle approach for managing chronic pain in particular. 

## 2. State of the Art Papers and Original Contributions

The Special Issue features state-of-the-art papers addressing key lifestyle factors of importance to patients having persistent pain and written by world leading experts and key opinion leaders in the field. For instance, an exciting state-of-the-art review proposes to clinicians working with patients with chronic pelvic pain to make use of the window of opportunity to prevent a potential transition from localized or periodic pain in the pelvis (e.g., pelvic pain during pregnancy and after delivery) towards persistent chronic pain, by promoting a healthy lifestyle [4]. In addition, original contributions to this Special Issue include literature reviews (systematic literature reviews with meta-analyses and narrative reviews) and exciting original research (trials, cohort studies, experimental lab work, and case–control studies) focussed on lifestyle and chronic pain. One important study reports that graded exposure in vivo treatment in patients with chronic low back pain and complex regional pain syndrome type I was accompanied by reductions in fear that preceded pain relief [5]. Another original study reported that opioid use was more closely related to perceived injustice and depression, but not anxiety and stress in 164 patients with chronic pain [6]. In a study of patients post COVID-19 infection (*n* = 567), more than 70% presented symptoms of central sensitisation [7]. The authors suggest that patient education and multimodal rehabilitation to target nociplastic pain can be considered in patients post COVID-19 infection with long-lasting symptoms of central sensitisation [8]. 

**Figure 1:**
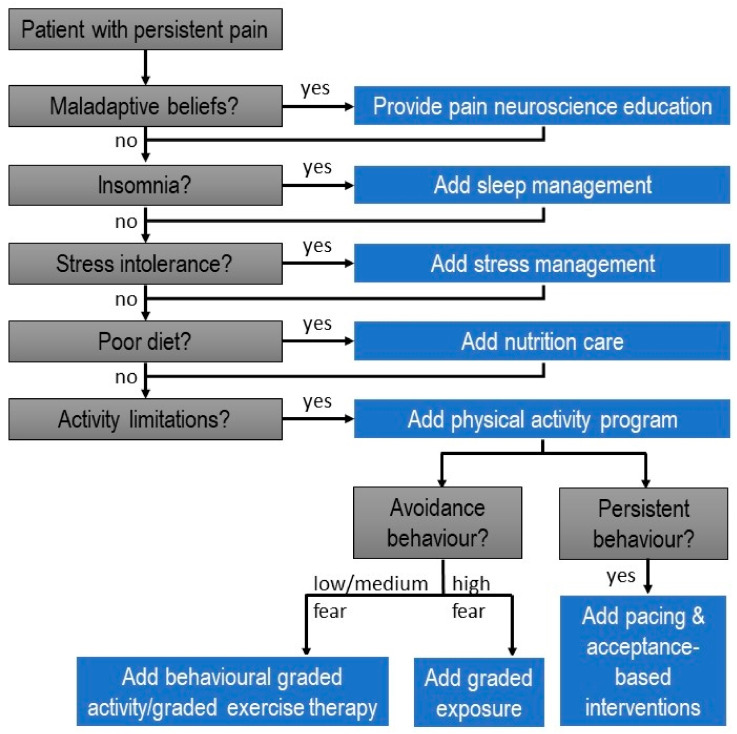
Towards precision medicine for chronic pain: clinical decision-making tree for providing an individually tailored lifestyle approach for patients with chronic pain.

In patients with episodic and chronic migraines, less mobility and less velocity of neck movements, without differences in muscle activity, were observed, with neck disability and kinesiophobia being negative and weakly associated with cervical movement [9]. This brings us to physical activity as a key lifestyle factor in patients with chronic pain, and the potential of exercise therapy and physical activity interventions to address this factor (Figure 1). A systematic literature review included in this Special Issue discusses the available evidence (including short- and long-term effects) supporting specific versus general exercise therapy for patients with chronic neck and shoulder pain [10].

Sleep is another key lifestyle factor perpetuating the condition in many patients with chronic pain [10,11]. This factor was addressed by several papers included in the Special Issue. First, a state-of-the-art review provided recommendations for best practices in the clinical assessment and treatment approaches to promote sleep health in children and adolescents with chronic pain [12]. Second, a systematic literature review with meta-analyses provides an overview of the associates of insomnia in people with chronic spinal pain, highlighting several significant associates of insomnia in this population [13]. This review is helpful in gaining a better understanding of the characteristics and potential origin of insomnia in patients with chronic spinal pain, including identifying patients with chronic spinal pain who are likely to have insomnia [13]. Finally, an original research report presents the cross-cultural translation and validation of the Pain and Sleep Questionnaire three-item index (PSQ-3) [14], allowing implementation of the PSQ-3 in Finland, potential leading to better understanding of the direct effects of pain on sleep in Finish patients with chronic pain [14].

Diet is another key lifestyle factor that is gaining scientific momentum in relation to chronic pain (treatment) [15,16]. Indeed, evidence supporting the role of diet as a perpetuating factor of chronic pain is cumulating [17,18,19,20,21,22]. This Special Issue contributes to this global move with several significant papers. First, an original contribution reported a study of 2367 middle-aged and older adults, and found that low protein intake and lack of regular exercise are associated with high odds for low back pain in women [23]. In addition, a review describes the current state of the art regarding nutrition in patients with chronic (non-cancer) pain, highlighting why nutrition is critical within a person-centred approach to pain management, and providing recommendations to guide clinicians in doing so [17]. In addition, another review included in this Special Issue focusses on patients with post-cancer pain, and argues that diet/nutrition might be ready to transition from a cancer recurrence/prevention strategy towards a chronic pain management modality for cancer survivors [24]. The importance of evidence-based pain management in cancer survivors is another global trend thoroughly addressed in this Special Issue, with another state-of-the-art review discussing how multiple modifiable lifestyle factors, such as stress, insomnia, diet, obesity, smoking, alcohol consumption and physical activity, play a role in shaping the pain experience after cancer, and how available treatment programs for cancer survivors can be improved by including an individually tailored lifestyle management approach [25].

## 3. Clinical Reasoning to Provide a Multimodal Lifestyle Approach

Together, this Special Issue contributes substantially to the paradigm shift towards a lifestyle approach for patients having non-ca-ncer and cancer-related chronic pain. In order to integrate the available evidence regarding lifestyle factors and chronic pain into clinical practice, clinicians can use the clinical decision-making tree for providing an individually tailored lifestyle approach for patients with chronic pain (displayed in Figure 1). It requires clinicians to screen for maladaptive beliefs as key determinants of an unhealthy lifestyle, and to address them accordingly, for instance by providing pain neuroscience education. In addition, the clinical decision-making tree guides clinicians through the main lifestyle factors for the chronic pain population, such as stress intolerance, poor sleep, poor diet and physical inactivity. If present, each of these lifestyle factors should be addressed during treatment. The resulting multimodal approach for managing patients with chronic pain implies tailoring treatment to the individual patient characteristics and therefore fits into the global move towards precision medicine [2]. Evidence supporting such a paradigm shift from a tissue- and disease-based approach towards individually tailored multimodal lifestyle interventions for chronic pain is mounting [4,12,23,25], but further study is needed. Several papers included in this Special Issue highlighted key areas for future research in this area (e.g., [4,25]). Such research efforts hold the potential to improve outcomes and decrease the psychological and socioeconomic burden of chronic pain.

## References

[1] Nijs J., D’Hondt E., Clarys P., Deliens T., Polli A., Malfliet A., Coppieters I., Willaert W., Tumkaya Yilmaz S., Elma Ö. (2020). Lifestyle and chronic pain across the lifespan: An inconvenient truth?. PM R J. Inj. Funct. Rehabil..

[2] National Research Council Committee on AFfDaNToD (2011). The National Academies Collection: Reports Funded by National Institutes of Health. Toward Precision Medicine: Building a Knowledge Network for Biomedical Research and a New Taxonomy of Disease.

[3] Nijs J., George S.Z., Clauw D.J., Fernández-de-las-Peñas C., Kosek E., Ickmans K., Fernández-Carnero J., Polli A., Kapreli E., Huysmans E. (2021). Central sensitisation in chronic pain conditions: Latest discoveries and their potential for precision medicine. Lancet Rheumatol..

[4] Gutke A., Sundfeldt K., De Baets L. (2021). Lifestyle and chronic pain in the pelvis: State of the art and future directions. J. Clin. Med..

[5] Bontinck J., den Hollander M., Kaas A.L., De Jong J.R., Timmers I. (2022). Individual patterns and temporal trajectories of changes in fear and pain during exposure in vivo: A multiple single-case experimental design in patients with chronic pain. J. Clin. Med..

[6] Kleinmann B., Wolter T. (2022). Opioid Consumption in chronic pain patients: Role of perceived injustice and other psychological and socioeconomic factors. J. Clin. Med..

[7] Goudman L., De Smedt A., Noppen M., Moens M. (2021). Is Central sensitisation the missing link of persisting symptoms after COVID-19 infection?. J. Clin. Med..

[8] Pinheiro C.F., Oliveira A.S., Will-Lemos T., Florencio L.L., Fernández-de-las-Peñas C., Dach F., Bevilaqua-Grossi D. (2021). Neck active movements assessment in women with episodic and chronic migraine. J. Clin. Med..

[9] Dueñas L., Aguilar-Rodríguez M., Voogt L., Lluch E., Struyf F., Mertens M.G., De Meulemeester K., Meeus M. (2021). Specific versus non-specific exercises for chronic neck or shoulder pain: A systematic review. J. Clin. Med..

[10] Nijs J., Mairesse O., Neu D., Leysen L., Danneels L., Cagnie B., Meeus M., Moens M., Ickmans K., Goubert D. (2018). Sleep disturbances in chronic pain: Neurobiology, assessment, and treatment in physical therapist practice. Phys. Ther..

[11] Campbell C.M., Buenaver L.F., Finan P., Bounds S.C., Redd M., McCauley L., Robinson M., Edw R.R., Smith M.T. (2015). Sleep Pain Catastrophizing and central sensitization in knee osteoarthritis patients with and without insomnia. Arthr. Care Res..

[12] Law E.F., Kim A., Ickmans K., Palermo T.M. (2022). Sleep health assessment and treatment in children and adolescents with chronic pain: State of the art and future directions. J. Clin. Med..

[13] Bilterys T., Siffain C., De Maeyer I., Van Looveren E., Mairesse O., Nijs J., Meeus M., Ickmans K., Cagnie B., Goubert D. (2021). Associates of insomnia in people with chronic spinal pain: A systematic review and meta-analysis. J. Clin. Med..

[14] Mikkonen J., Leinonen V., Luomajoki H., Kaski D., Kupari S., Tarvainen M., Selander T., Airaksinen O. (2021). Cross-cultural adaptation, reliability, and psychophysical validation of the pain and sleep questionnaire three-item index in finnish. J. Clin. Med..

[15] Elma Ö., Yilmaz S.T., Deliens T., Coppieters I., Clarys P., Nijs J., Malfliet A. (2020). Do nutritional factors interact with chronic musculoskeletal pain? A systematic review. J. Clin. Med..

[16] Nijs J., Tumkaya Yilmaz S., Elma Ö., Tatta J., Mullie P., Vanderweeën L., Clarys P., Deliens T., Coppieters I., Weltens N. (2020). Nutritional intervention in chronic pain: An innovative way of targeting central nervous system sensitization?. Expert Opin. Ther. Targets.

[17] Brain K., Burrows T.L., Bruggink L., Malfliet A., Hayes C., Hodson F.J., Collins C.E. (2021). Diet and Chronic Non-Cancer Pain: The State of the Art and Future Directions. J. Clin. Med..

[18] Correa-Rodríguez M., Casas-Barragán A., González-Jiménez E., Schmidt-RioValle J., Molina F., Aguilar-Ferrándiz M.E. (2020). Dietary inflammatory index scores are associated with pressure pain hypersensitivity in women with fibromyalgia. Pain Med..

[19] Brain K., Burrows T.L., Rollo M.E., Chai L.K., Clarke E.D., Hayes C., Hodson F.J., Collins C.E. (2019). A systematic review and meta-analysis of nutrition interventions for chronic noncancer pain. J. Human Nutr. Dietet..

[20] Du C., Smith A., Avalos M., South S., Crabtree K., Wang W., Kwon Y.H., Vijayagopal P., Juma S. (2019). Blueberries Improve Pain, Gait Performance, and Inflammation in Individuals with Symptomatic Knee Osteoarthritis. Nutrients.

[21] Elma Ö., Yilmaz S.T., Deliens T., Coppieters I., Clarys P., Nijs J., Malfliet A. (2020). Nutritional factors in chronic musculoskeletal pain: Unravelling the underlying mechanisms. Br. J. Anaesthes..

[22] Tatta J., Nijs J., Elma Ö., Malfliet A., Magnusson D. (2022). The Critical Role of Nutrition Care to Improve Pain Management: A Global Call to Action for Physical Therapist Practice. Phys. Ther..

[23] Noh H.M., Choi Y.H., Lee S.K., Song H.J., Park Y.S., Kim N., Cho J. (2022). Association between dietary protein intake, regular exercise, and low back pain among middle-aged and older korean adults without osteoarthritis of the lumbar spine. J. Clin. Med..

[24] Tümkaya Yılmaz S., Malfliet A., Elma Ö., Deliens T., Nijs J., Clarys P., De Groef A., Coppieters I. (2022). Diet/Nutrition: Ready to transition from a cancer recurrence/prevention strategy to a chronic pain management modality for cancer survivors?. J. Clin. Med..

[25] Lahousse A., Roose E., Leysen L., Yilmaz S.T., Mostaqim K., Reis F., Rheel E., Beckwée D., Nijs J. (2022). Lifestyle and pain following cancer: State-of-the-art and future directions. J. Clin. Med..

